# Anti-TNF-α Drugs Differently Affect the TNFα-sTNFR System and Monocyte Subsets in Patients with Psoriasis

**DOI:** 10.1371/journal.pone.0167757

**Published:** 2016-12-09

**Authors:** Lara Gibellini, Sara De Biasi, Elena Bianchini, Regina Bartolomeo, Antonella Fabiano, Marco Manfredini, Federica Ferrari, Giuseppe Albertini, Tommaso Trenti, Milena Nasi, Marcello Pinti, Anna Iannone, Carlo Salvarani, Andrea Cossarizza, Giovanni Pellacani

**Affiliations:** 1 Department of Surgery, Medicine, Dentistry and Morphological Sciences, University of Modena and Reggio Emilia, Modena, Italy; 2 Department of Life Sciences, University of Modena and Reggio Emilia, Modena, Italy; 3 Dermatologic Unit, IRCCS - Santa Maria Nuova Hospital, Reggio Emilia, Italy; 4 Department of Clinical Pathology - NOCSAE Baggiovara, Modena, Italy; 5 Department of Diagnostics, Clinical and Public Health Medicine, University of Modena and Reggio Emilia, Modena, Italy; 6 Reumatology Unit, IRCCS - Santa Maria Nuova Hospital, Reggio Emilia, Italy; 7 Department of Medical and Surgical Sciences for Children and Adults, University of Modena and Reggio Emilia, Modena, Italy; Institut Cochin, FRANCE

## Abstract

TNF-α has a central role in the development and maintenance of psoriatic plaques, and its serum levels correlate with disease activity. Anti-TNF-α drugs are, however, ineffective in a relevant percentage of patients for reasons that are currently unknown. To understand whether the response to anti-TNF-α drugs is influenced by the production of anti-drug antibodies or by the modulation of the TNFα-TNFα receptor system, and to identify changes in monocyte phenotype and activity, we analysed 119 psoriatic patients who either responded or did not respond to different anti-TNF-α therapies (adalimumab, etanercept or infliximab), and measured plasma levels of TNF-α, TNF-α soluble receptors, drug and anti-drug antibodies. Moreover, we analyzed the production of TNF-α and TNF-α soluble receptors by peripheral blood mononuclear cells (PBMCs), and characterized different monocyte populations. We found that: i) the drug levels varied between responders and non-responders; ii) anti-infliximab antibodies were present in 15% of infliximab-treated patients, while anti-etanercept or anti-adalimumab antibodies were never detected; iii) plasma TNF-α levels were higher in patients treated with etanercept compared to patients treated with adalimumab or infliximab; iv) PBMCs from patients responding to adalimumab and etanercept produced more TNF-α and sTNFRII *in vitro* than patients responding to infliximab; v) PBMCs from patients not responding to infliximab produce higher levels of TNF-α and sTNFRII than patients responding to infliximab; vi) anti- TNF-α drugs significantly altered monocyte subsets. A complex remodelling of the TNFα-TNFα receptor system thus takes place in patients treated with anti-TNF-α drugs, that involves either the production of anti-drug antibodies or the modulation of monocyte phenotype or inflammatory activity.

## Introduction

Psoriasis, also defined as plaque psoriasis or *psoriasis vulgaris*, is a chronic inflammatory disease characterized by the infiltration of inflammatory cells in the skin and hyper-proliferation of keratinocytes, resulting in red-coloured plaques, mainly located on elbows, knees, scalp and in the sacral area [1–3]. Although the molecular mechanisms driving the pathogenesis of psoriasis is still unknown, both immune system dysregulation and environmental factors, including diet, psychosocial stress, certain drugs and infections, are critically involved [4–7]. The main immune cells involved in psoriasis are myeloid dendritic cells (mDCs) and T lymphocytes. Myeloid DCs sustain the activation and differentiation of several T cell subsets, including type-1 helper T cells (Th1), type-17 helper T cells (Th17), and γδ T cells. These cells secrete a number of specific cytokines, including tumour necrosis factor (TNF)-α, interferon (IFN)-γ, interleukin (IL)-1, IL-6, IL-17A, IL-17F and IL-22, which play a role in the pathogenesis of psoriasis [3, 8].

Among these cytokines, TNF-α is a crucial pathogenetic driver. TNF-α binds two different cell-membrane receptors, TNF receptor (TNFR) type 1, also known as p55, and TNFR2, also known as p75. Soluble forms of these receptors, namely soluble TNFR1 (sTNFR1) and soluble TNFR2 (sTNFR2), have also been identified and likely act as endogenous TNF-α inhibitors. Increased levels of sTNFR1 have been found in serum from patients with psoriasis [9]. TNF-α modulates the activity of various cells. Firstly, it induces the maturation of DCs, and skews monocyte differentiation from macrophages to DCs, thus favouring adaptive immunity [10]. Secondly, it promotes the expression of adhesion molecules, including intercellular adhesion molecule-1 (ICAM-1), vascular cell adhesion molecule-1 (VCAM-1) and E-selectin, on dermal vascular endothelial cells. Thirdly, it induces the expression of chemokines, such as chemokine ligand (CCL)-20, CCL5, and CCL2 on keratinocytes and dermal fibroblasts [11].

Several TNF-α blockers, including neutralizing antibodies and soluble TNF-α receptors, have been developed, and are currently used in clinical practice. Adalimumab, etanercept and infliximab represent the first-generation inhibitors targeting TNF-α, and are largely used in clinical settings. Adalimumab is a fully human monoclonal antibody, while infliximab is a chimeric human/mouse monoclonal antibody containing almost 75% human-derived amino acids and almost 25% mouse-derived amino acids. Etanercept is a fusion protein where a dimer of the extracellular domains of human TNFR2 is fused to the Fc portion of human IgG1 [12].

Anti-TNF-α drugs provide remission in a great proportion of patients, reducing inflammation and decreasing the “Psoriasis Area and Severity Index” (PASI) [13–15]. However, a relevant percentage of patients fails to respond to treatment [16, 17]. The mechanisms driving the different clinical responses to anti-TNF-α drugs are still unknown, but are a crucial issue considering the high cost of these drugs and the risk of rare but severe adverse events. Several mechanisms have been proposed to explain the different responses, including i) the presence of genetic polymorphisms in genes encoding proteins of the TNF-α system [18, 19], and ii) the formation of neutralising and non-neutralising anti-drug antibodies (ADAs) [20]. We aimed to investigate whether anti-TNF-α drugs impair the functional capacity of PBMCs to respond to lipopolysaccharide (LPS) and/or induce modifications among the main populations of monocytes, which are the major producers of TNF-α. To address this question, we quantified plasma levels of ADAs, TNF-α, sTNFRI and sTNFRII, examined the functional capacity of PBMCs to respond to lipopolysaccharide (LPS), and characterized the main populations of monocytes in a large cohort of patients with psoriasis treated with different biological drugs (anti-TNF), including responders and non-responders to treatment.

## Methods

### Study design and clinical cohort

A total of 119 patients with psoriasis and psoriatic arthritis were enrolled at the Department of Dermatology of the University of Modena and Reggio Emilia (Modena, Italy). Of these, 48 were treated with adalimumab (standard dosage for psoriasis), 30 with etanercept (standard dosage for psoriasis), 41 with infliximab (standard dosage for psoriasis). Twenty healthy controls (HC, median age 50.1, range 26–69) were also enrolled. Patients’ demographic and clinical characteristics are shown in Table 1. In each group, on the basis of the PASI, patients were divided into two subgroups: responders (R) and non-responders (NR). R were defined as having a 75% reduction in PASI compared to baseline (PASI 75) within six months of treatment. NR were those who did not achieve a 50% reduction in PASI compared to baseline within six months of treatment. Patients achieving a response comprised between 50% and 75% in PASI were included in the NR group. Ninety-five percent of patients enrolled in this study had been suffering from psoriasis for more than five years. Moreover, according to national and international guidelines for the treatment of psoriasis, therapies based on biologics are restricted to patients with moderate to severe psoriasis. Therefore, before the beginning of treatment, the mean baseline PASI was 16.42 (SD: 3.73). Given the long and relapsing nature of the disease, 35.3% of patients were receiving their first biological drug, whereas 64.7% had been previously treated with other biologics and then switched to the current treatment for at least 6 months. Patients could be included if they were 18 years of age or older, if they did not present any relevant autoimmune comorbidities, if they had not received prior treatment with anti-TNF drugs or if the last drug treatment lasted at least six months, and if they had suspended treatment in the last four months before sampling. Enrolment of patients started on August 26^th^ 2014 and ended on April 20^th^ 2015. Blood samples were collected during the follow-up visits of patients receiving biological drugs. For patients treated with etanercept or adalimumab, blood samples were obtained three months after the last drug injection, while for patients treated with infliximab, blood samples were obtained two months after the last drug infusion. The study was approved by the local Ethical Committee of Modena (identification code number 296/13) and by the Institutional Review Board of the Dept. of Surgery, Medicine, Dentistry and Morphological Sciences of the University of Modena and Reggio Emilia, and was conducted according to the Declaration of Helsinki principles. Signed consent forms were collected from all patients.

**Table 1:** Demographic and clinical characteristics of patients.

	Adalimumab (n = 48)	Etanercept (n = 30)	Infliximab (n = 41)
Median age, years (range)	54 (26–81)	54 (24–82)	53 (29–80)
Gender, % male	62.5	86.7	82.9
Therapy effectiveness, % responders	62.5	66.7	80.8
Psoriatic arthritis, % presence	83.3	56.6	56.0
Received previous biologic therapy, %	62.5	40.0	85.3

Twenty ml of venous blood were collected from each patient into ethylenediamine-tetraacetic acid (EDTA) tubes. Samples were centrifuged and plasma was stored at -80°C. Peripheral blood mononuclear cells (PBMCs) were isolated by Ficoll-Hypaque density gradient according to standard procedures and stored in liquid nitrogen until use.

### Quantification of plasma levels of TNF-α, sTNFRI, sTNFRII

The plasma levels of TNF-α, sTNFRI, sTNFRII were quantified by Quantikine ELISA kit (R&D Systems, Minneapolis, MN, USA), following instructions provided by the manufacturer. In particular, the following kits were used: human TNF-α Quantikine HS ELISA kit (catalog number HSDTA00D), human sTNFRI/TNFRSF1A Quantikine ELISA Kit (catalog number DRT100), and human sTNFRII/TNFRSF1B Quantikine ELISA Kit (catalog number DRT200).

### Quantification of plasma levels of etanercept, anti-etanercept antibodies, adalimumab, anti-adalimumab antibodies, infliximab and anti-infliximab antibodies

The plasma levels of etanercept, anti-etanercept antibodies, adalimumab, anti-adalimumab antibodies, infliximab and anti-infliximab antibodies were quantified by the following ELISA kits: shikari Q-ETA, shikari S-ATE, shikari Q-ADA, shikari S-ATA, shikari Q-INFLIXI and shikari Q-ATI (all from Matriks Biotechnology, Ankara, Turkey).

### Stimulation of PBMCs with lipopolysaccharide (LPS)

PBMCs were thawed and plated in RPMI-1640 complete medium (10% foetal bovine serum, 1% penicillin/streptomycin, 2 mM L-glutamine; all from Life Technologies Corporation, Carlsbad, CA, USA) with or without 50 ng/mL LPS (Sigma Aldrich, St. Louis, MO, USA) for 16 hours. Supernatant was collected and used for the quantification of TNF-α, sTNFRI and sTNFRII by human TNF-α Quantikine ELISA kit (catalog number DTA00C), human sTNFRI/TNFRSF1A Quantikine ELISA Kit and human sTNFRII/TNFRSF1B Quantikine ELISA Kit.

### Flow cytometric analysis of monocytes

PBMCs were thawed according to standard procedures [21]. Monocytes were identified on the basis of their size, granularity and expression of HLA-DR and CD14, by using polychromatic flow cytometry. Living cells were identified for the negativity to Red fixable live dead (Thermofisher, Eugene, OR, USA). Then, three populations of monocytes were identified on the basis of anti-CD14 APC (Becton Dickinson, San José, CA, USA) and -CD16 FITC (BD) expression. The expression of HLA-DR (PE-conjugated, BD) was evaluated among different monocyte subpopulation. Anti-CD3-V450, anti-CD19-V450 and anti-CD56-V450 were used for the DUMP channel. Samples were washed twice and immediately analysed by using an Attune Nxt Acoustic Focusing Cytometer (Thermofisher). The instrument is equipped with a blue laser (488 nm), a red laser (637 nm), a yellow laser (561 nm), and a violet laser (405 nm). Data were acquired in the list mode using Attune Cytometric 2.1 software and then analysed by FlowJo 9.9 (Tree Star Inc, Ashland, OR, USA) under Mac OSX [22].

### Statistical analysis

Kruskal-Wallis test with Dunn’s Multiple Comparison Test was used to compare TNF-α, sTNFRI and sTNFRII plasma levels in patients from different groups. Mann-Whitney test was used to compare drug concentration among different groups. All statistical analyses were performed by using Prism 5.0 software (GraphPad, La Jolla, CA, USA); a *p*-value <0.05 was considered significant [23]. Principal component analysis was performed by using MatLab software, version R2013a.

## Results

### Patient characteristics and clinical response

We studied 119 patients receiving biological drugs, whose plasma was screened for TNF-α, sTNFRI and sTNFRII, drug and anti-drug antibodies levels. Among patients treated with adalimumab (mean age: 54 years, range: 26–81), 30 were R (62.5%) and 18 were NR (37.5%); among patients treated with etanercept (mean age: 54, range: 24–82), 20 were R (66.7%) and 10 were NR (33.3%); among patients treated with infliximab (mean age: 53, range: 29–80), 29 were R (70.8%) and 12 were NR (29.2%).

### Plasma levels of drugs and anti-drug antibodies

**Fig 1:**
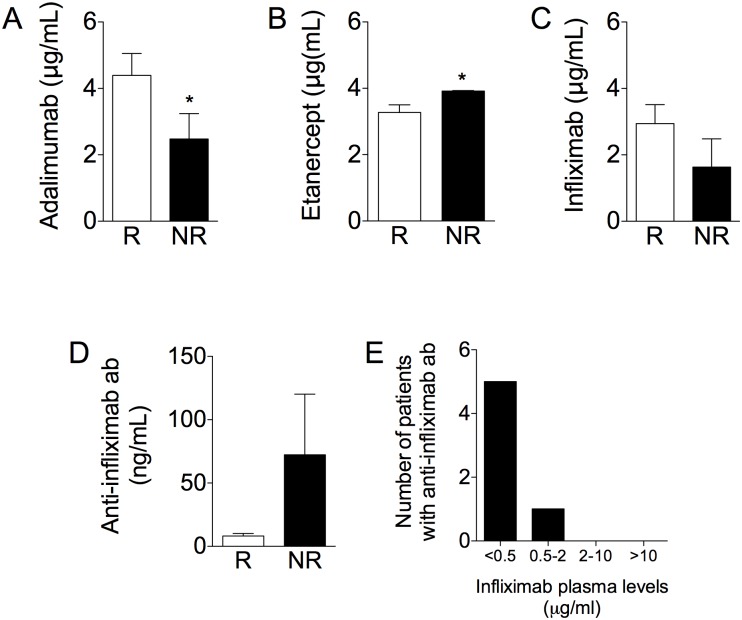
Anti-TNF-α plasma level and anti-drug antibodies. (A) Adalimumab concentration in plasma from patients with psoriasis who respond (R) or do not respond (NR) to therapy. *p<0.05. (B) Etanercept concentration in plasma from patients with psoriasis who respond (responders, R) or do not respond (non responders, NR) to therapy. *p<0.05. (C) Infliximab concentration in plasma from R or NR to therapy. (D) Anti-infliximab antibodies concentration in plasma from R (n = 3) or NR (n = 3) to therapy. (E) Number of patients with anti-infliximab antibodies stratified according to infliximab plasma levels.

As shown in Fig 1A, the mean plasma concentration of adalimumab in R was 4.39 μg mL^-1^ (range: 0.002–9.89), and a significant difference was observed between R and NR (4.39±0.6 vs. 2.47±0.76 μg mL^-1^, p<0.05). Concerning etanercept, the mean concentration of the drug was higher in NR (3.91±0.01 μg mL^-1^) than in R (3.27±0.23 μg mL^-1^, p<0.05, Fig 1B). In patients treated with infliximab, the mean concentration of the drug was 2.63 μg mL^-1^ (range: 0.04–9.29 μg mL^-1^) in R, and 1.63 μg mL^-1^ in NR (range: 0.04–7.14 μg mL^-1^), and no significant differences were observed between R and NR (Fig 1C).

Studies in the literature have shown that ADA for adalimumab are often present in patients assuming this drug. In our study, while anti-drug antibodies were undetectable in patients treated with adalimumab and etanercept, anti-drug antibodies were detected in the plasma of 6 out of 38 (15.8%) patients treated with infliximab (Fig 1D). No significant difference was found among R and NR, and in most patients (5 out of 6) plasma levels of infliximab were <0.5 μg mL^-1^ (Fig 1E).

### Plasma levels of TNF-α, sTNFRI and sTNFRII

**Fig 2:**
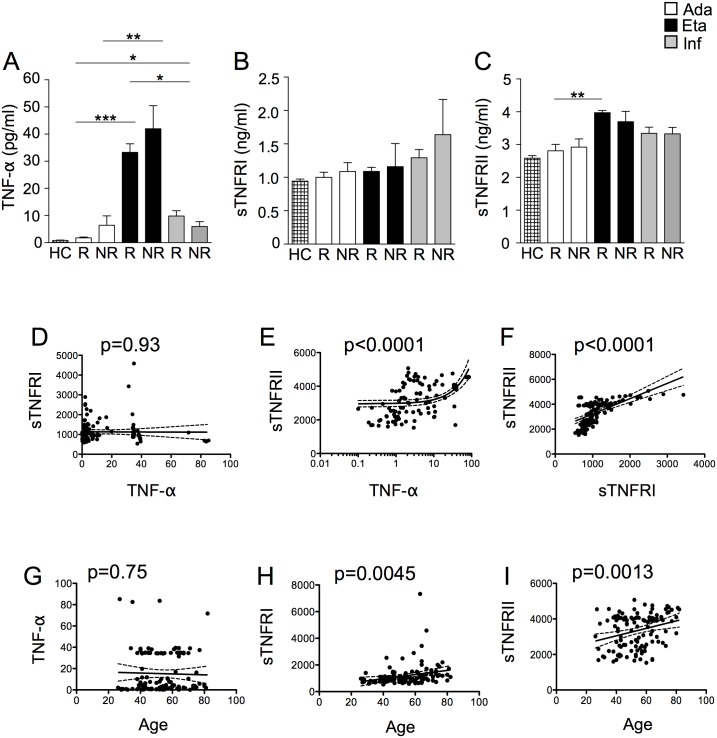
Patients treated with etanercept have higher plasma levels of TNF-α. (A) TNF-α concentrations in plasma from healthy controls (HC), and from psoriatic patients who were R or NR to adalimumab, etanercept or infliximab. The number of patients (n) was 29 for R to adalimumab, 16 for NR to adalimumab, 24 for R to etanercept, 12 for NR to etanercept, 30 for R to infliximab, 11 for NR to infliximab, 20 for HC. *p<0.05; **p<0.01; ***p<0.001. (B) sTNFRI concentrations in plasma from HC, and from psoriatic patients who were R or NR to adalimumab, etanercept or infliximab. The number of patients for each group is the same as in panel A. (C) sTNFRII concentrations in plasma from HC, and from psoriatic patients who were R or NR to adalimumab, etanercept or infliximab. The number of patients for each group is the same as in panel A. (D) Linear regression analysis for the correlation between plasma levels of TNF-α and plasma levels of sTNFRI. (E) Linear regression analysis for the correlation between plasma levels of TNF-α and plasma levels of sTNFRII, (F) Linear regression analysis for the correlation between plasma levels of sTNFRI and plasma levels of sTNFRII. (G) Linear regression analysis for the correlation between age and plasma levels of TNF-α. (H) Linear regression analysis for the correlation between age and plasma levels of sTNFRI. (I) Linear regression analysis for the correlation between age and plasma levels of sTNFRII.

**Fig 3:**
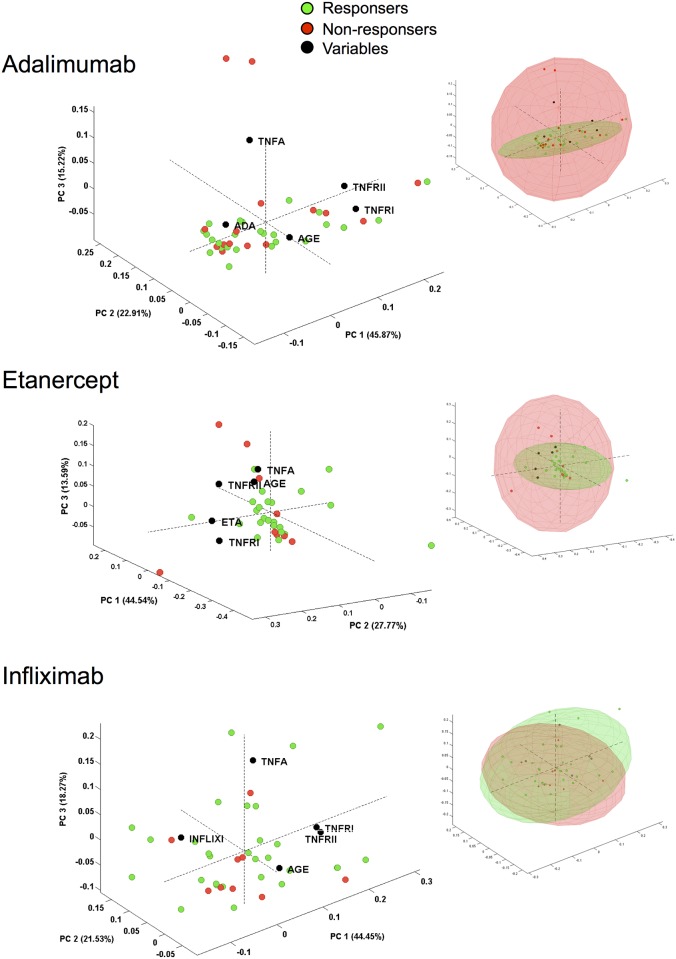
Principal component analysis bi-plot (loadings and scores). The spatial distribution of the immunological parameters and of R and NR to different treatments is based on the first three principal components (PC). Each dot represents a single patient. Confidential ellipses are also shown. TNFA, plasma levels of TNF-α; TNFRI, plasma levels of sTNFRI; TNFRII, plasma levels of sTNFRII; ADA, plasma levels of adalimumab; ETA, plasma levels of etanercept; INFLIXI, plasma levels of infliximab.

Plasma levels of TNF-α, sTNFRI and sTNFRII were quantified by ELISA. Results concerning plasma levels of TNF-α, sTNFRI and sTNFRII are reported in Fig 2A–2C. To have reference value, TNF-α, sTNFRI and sTNFRII levels were also quantified in 20 HC. TNF-α sTNFRI, and sTNFRII levels in HC were 0.83±0.14 pg mL^-1^, 942.2±31.77 ng mL^-1^ and 2,587±75.85 ng mL^-1^. TNF-α concentration in plasma from patients treated with etanercept was significantly higher compared to those treated with adalimumab or infliximab. Patients responding to etanercept had higher levels of TNF-α compared to patients responding to adalimumab or infliximab. No differences were detected between R and NR to the different treatments. Levels of sTNFRI or sTNFRII were similar among all groups of patients (Fig 2B and 2C). As shown in Fig 2D, no correlation between plasma levels of TNF-α and plasma levels of sTNFRI was observed. Conversely, a correlation was present between plasma levels of TNF-α and plasma levels of sTNFRI, as well as between plasma of sTNFRI and sTNFRII (Fig 2E and 2F). We also found that, among all patients, plasma levels of sTNFRI and sTNFRII, but not TNF-α, correlate with age (Fig 2G–2I). Explorative principal component analysis on immunologic parameters revealed that R and NR to the different treatments did not form distinct clusters (Fig 3).

### *In vitro* activity of PBMCs from patients

**Fig 4:**
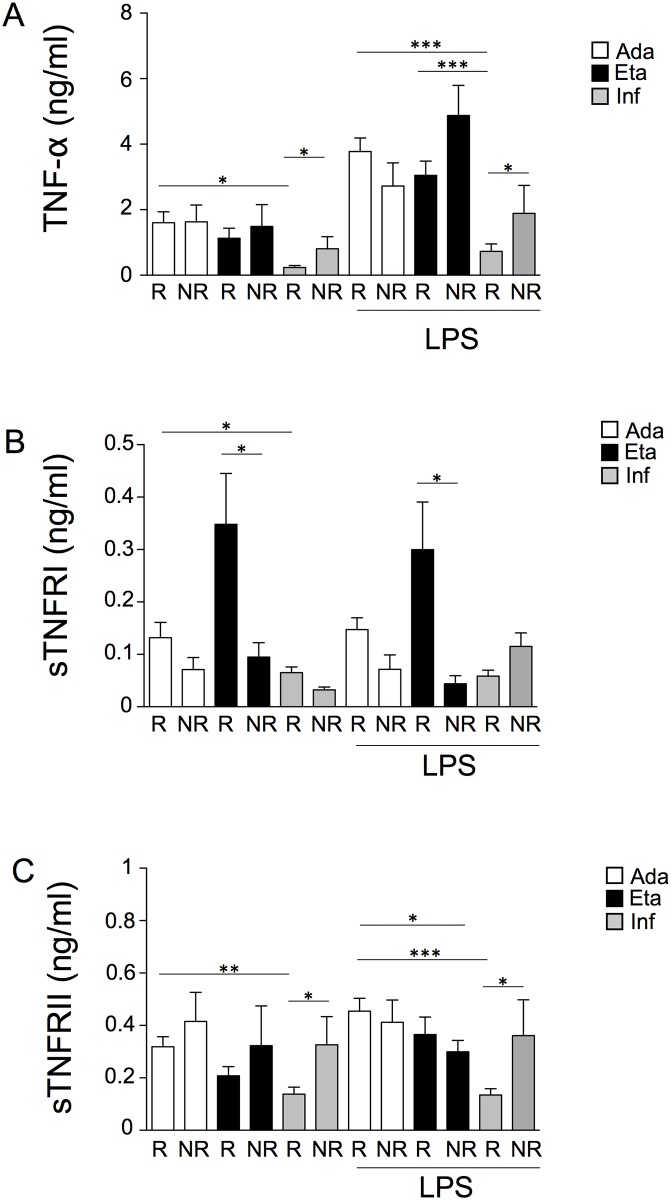
PBMCs from patients responding to adalimumab and etanercept produce higher levels of TNF-α and sTNFRII than PBMCs from patients responding to infliximab. (A) TNF-α levels were quantified in the supernatant of peripheral blood mononuclear cells (PBMCs) in the absence or in the presence of lipopolysaccharide (LPS) for 24 hours. PBMCs were obtained from patients treated with adalimumab, etanercept or infliximab. Ada, adalimumab; Eta, etanercept; Inf, infliximab; NR, non-responders; R, responders. *p<0.05; ***p<0.001. (B) sTNFRI levels were quantified in the supernatant of PBMCs in the absence or in the presence of LPS for 24 hours. PBMCs were obtained from patients treated with Ada, Eta or Inf. *p<0.05. (C) sTNFRII levels were quantified in the supernatant of PBMCs in the absence or in the presence of LPS for 24 hours. PBMCs were obtained from patients treated with Ada, Eta or Inf. *p<0.05; **p<0.01; ***p<0.001.

In order to evaluate whether anti-TNF-α drugs affected the spontaneous production of cytokines in PMBCs together with their capacity to respond to LPS, cells were cultured in the presence of LPS for 16 hours, and supernatants were assayed for the production of TNF-α, sTNFRI and sTNFRII. Results are reported in Fig 4A–4C. Interestingly, PBMCs from patients not responding to infliximab produce higher levels of TNF-α than PBMCs obtained from patients responding to treatment with infliximab. Moreover, PBMCs obtained from patients responding to adalimumab or etanercept produced higher levels of TNF-α than PBMCs from infliximab-treated patients, at the basal levels or in the presence of LPS.

Concerning sTNFRI, PBMCs obtained from patients responding to adalimumab produced higher levels of sTNFRI than PBMCs from patients responding to infliximab. PBMCs obtained from patients treated with etanercept and not responding to therapy produce lower levels of sTNFRI than PBMCs from R. Concerning sTNFRII, PBMCs obtained from patients responding to adalimumab produced higher levels of sTNFRII than PBMCs from infliximab-treated patients, at the basal levels or after LPS stimulation. PBMCs from patients responding to etanercept produced higher levels of sTNFRII than PBMCs from patients treated with infliximab, only in the presence of LPS. PBMCs from patients not responding to infliximab produce almost two-fold levels of sTNFRII than PBMCs obtained from patients treated with infliximab and responding to the drug.

### Quantification of monocytes and expression of HLA-DR and CD14 surface markers

Monocytes represent a heterogeneous cell population that express MHC class II molecules (*i*.*e*., HLA-DR) and can be divided into three different subsets on the basis of CD14 and CD16 expression. Classical monocytes express CD14 and do not express CD16 (CD14++,CD16-), non-classical monocytes express CD14 and high levels CD16 (CD14+,CD16++), and intermediate monocytes express high levels of CD14 and CD16 (CD14++,CD16+) [24]. Different subsets of monocytes are associated with a different ability to present antigens, as well as to different levels of proinflammatory cytokines and expression of homing receptors.

**Fig 5:**
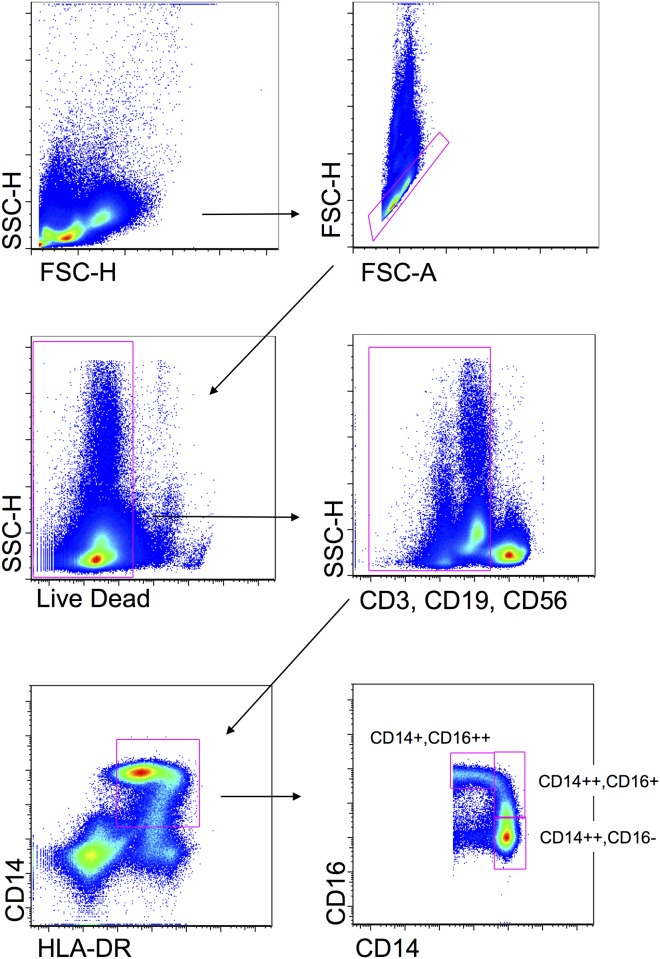
Gating strategy used to identify monocyte subsets among PBMCs. Monocytes were first selected on the basis of their physical parameters, i.e. forward scatter (FSC) and side scatter (SSC). Doublets were eliminated from the analysis (upper right panel). Then, living monocyes were selected according to the negativity to Live Dead probe (middle left panel). Cells negative for the DUMP channel (anti-CD3, anti-CD19 and anti-CD56) were then selected (middle right panel). Among these, monocytes were selected according to positivity to CD14 and HLA-DR (lower left panel). In this population, three monocyte subsets were identified on the basis of the expression of CD14 and CD16 (lower right panel).

**Fig 6:**
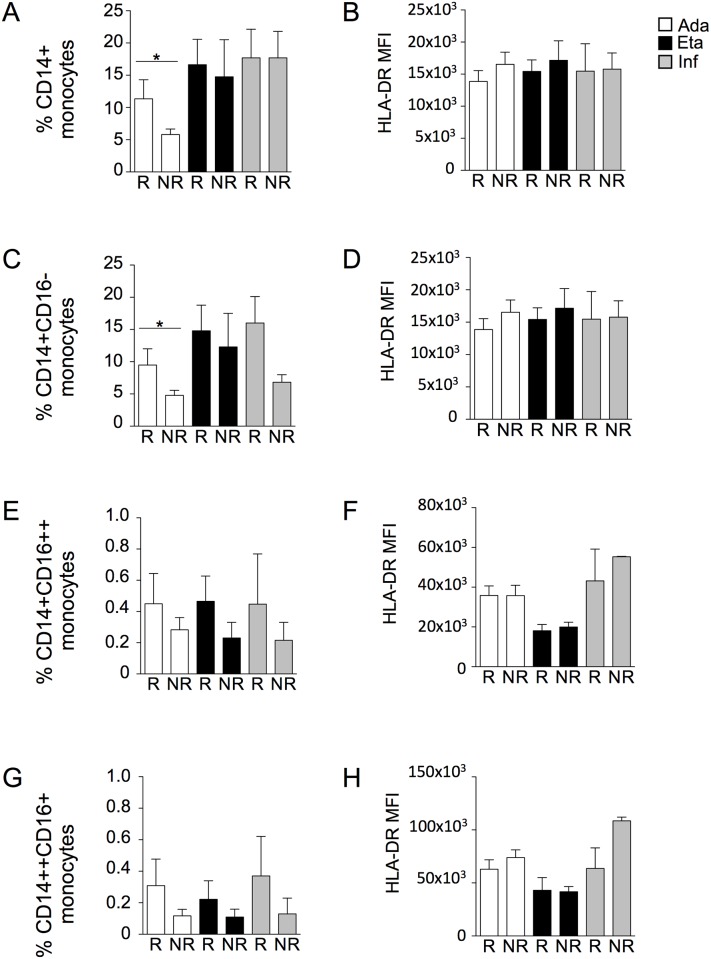
Adalimumab affects the distribution of monocyte subsets. (A) Percentage of CD14+ monocytes (*i*.*e*., all monocytes) in patients treated with adalimumab (Ada), etanercept (Eta) or infliximab (Inf). *p<0.05. (B) Quantification of HLA-DR expression in CD14+ monocytes. (C) Percentage of CD14++,CD16- (classical) monocytes in patients treated with Ada, Eta or Inf. *p<0.05. (D) Quantification of HLA-DR expression in CD14++,CD16- monocytes. (E) Percentage of CD14+,CD16++ (non-classical) monocytes in patients treated with Ada, Eta or Inf. (F) Quantification of HLA-DR expression in CD14+,CD16++ monocytes. (G) Percentage of CD14++,CD16+ (intermediate) monocytes in patients treated with Ada, Eta or Inf. (H) Quantification of HLA-DR expression in CD14++,CD16+ monocytes. For all panels: R, responders; NR, non-responders; MFI, median fluorescence intensity.

Given the importance of monocytes in inflammatory diseases, we identified these cells and their subsets in PBMCs from patients treated with different anti-TNF-α drugs. As shown in Fig 5, monocytes were first identified according to forward scatter (FSC) and side scatter (SSC) (upper left panel). Then, doublets were excluded (upper right panel), and living cells were selected for negativity to Live Dead probe (middle left panel). Cells proving positive for CD3, CD19 and CD56 were then excluded (middle right panel), and true monocytes were identified on the basis of the positivity for the expression of MHC-class II molecules and CD14 (lower left panel). In this population, three different monocyte subsets were identified by flow cytometry on the basis the expression of CD14 and CD16 (lower right panel), as described [25]. Then, to analyse the activation status of these monocyte subsets, the expression of HLA-DR was quantified in each group. The percentage of monocyte subsets is reported in Fig 6A, 6C, 6E and 6G, and the quantification of HLA-DR in each subset is reported in Fig 6B, 6D, 6F and 6H. We found that the percentage of CD14+, as wells as the percentage of CD14++,CD16- (*i*.*e*. classical) monocytes, was higher in patients responding to adalimumab than in patients not responding to the same drug. A slight decrease in the percentage of classical monocytes, although not significant, was observed in patients not responding to infliximab. Concerning HLA-DR, its expression was slightly higher in non-classical and intermediate monocytes from patients treated with infliximab or adalimumab compared to patients treated with etanercept.

## Discussion

The hallmark of psoriasis is an extensive inflammation affecting the skin, mainly mediated by Th1/Th17 cytokines, with a key role of TNF-α [26, 27]. For this reason, TNF-α blockers are used in clinical practice in patients who fail to respond to conventional therapies. A number of clinical trials and observational studies have shown that anti-TNF-α drugs are effective in the treatment of psoriasis, as they reduce inflammation in psoriatic lesions and reverse disease activity. However, the effectiveness of therapies based on anti-TNF-α drugs is only partial, as a relevant percentage of patients fail to respond to this therapy, and maintain an active disease. Therefore, presently, it is a major challenge to understand the molecular mechanisms at the basis of the different responses to treatments.

We found that 15.8% of patients treated with infliximab had detectable levels of ADAs, most of them being associated with low plasma infliximab concentrations. The presence of anti-infliximab antibodies (ATIs) has also been described in patients with Crohn’s disease (CD) or rheumatoid arthritis (RA), with a prevalence ranging from 12% to 44% in CD, and 6–61% in RA [20, 26, 28]. According to a recent systematic review, antibodies against infliximab, etanercept and adalimumab were reported in 5.4–43.6%, 0–18.3% and 6–45% of patients with psoriasis, respectively [29]. Regarding ATIs concentrations, we did not observe a significant statistical difference among R and NR. However, while the concentration range of ATIs was 5.4–63.1 ng/ml for R, for NR it was 13.8–422.45 ng/ml, suggesting that a higher concentration of ATIs could be involved in therapy ineffectiveness.

Concerning plasma levels of TNF-α, sTNFRI and sTNFRII, the main differences involved TNF-α levels. Firstly, we found that TNF-α plasma levels do not correlate with the subjects’ age. This was quite unexpected, as in the healthy population TNF-α plasma levels increase with age [30]. In psoriatic patients, the absence of correlation is probably due to the removal of TNF-α by the drugs. Conversely, levels of sTNFRI and sTNFRII correlate with patients’ age. Concerning the different drugs, patients treated with etanercept, both R or NR, display higher levels of TNF-α compared to patients treated with adalimumab or infliximab. This was unexpected, since anti-TNF-α therapy causes a decrease in the frequencies of pro-inflammatory T helper cells, including Th1 and Th17, and a concomitant decline in their associated cytokines, principally TNF-α. The more obvious and possible explanation is related to the test that was used to detect TNF-α in plasma, that could most likely be able to detect both free TNF-α and TNF-α bound to etanercept. However, whether or not TNF-α was detected by the assay in its free or bound form, the total concentration of TNF-α in patients treated with etanercept was higher. Although explorative principal component analysis on immunologic and haematological parameters did not reveal any particular cluster of patients, the production of TNF-α and its soluble receptors was altered when PBMCs were cultured *in vitro*, in the presence or in the absence of LPS. When stimulated with LPS, PBMCs from patients responding to etanercept or adalimumab produce almost 4-fold TNF-α together with sTNFRII than PMBCs from patients receiving infliximab. PBMCs from patients not responding to infliximab produced higher levels of TNF-α and sTNFRII, whereas PBMCs from patients treated with etanercept and not responding to this therapy, produced lower levels of sTNFRI. This could partially explain the higher TNF-α levels observed in these patients’ plasma. Previous studies suggested that etanercept and infliximab strongly affect gene expression in PBMCs, and that effects of etanercept were different from infliximab in the modulation of proinflammatory genes [31, 32]. Our data are thus in agreement with above-said observations.

We found that anti-TNF-α drugs also modify monocyte subsets in patients with psoriasis. Circulating monocytes derive from the bone marrow and mediate both regulatory and effector functions in adaptive and innate immunity. In response to several stimuli, monocytes can migrate into tissues where they can differentiate into phagocytes, dendritic cells and osteoclasts [33]. Three major subsets of circulating monocytes have been identified so far, that differ in the expression of chemokine receptors, migratory properties, reactive oxygen species generation, and ability to differentiate into effector cells [34]. The frequency and distribution of monocyte subsets have been analysed in a variety of inflammatory diseases, including RA, CD and ulcerative colitis, and in certain cases represent a suitable marker of response to drugs [35–37]. For instance, the absolute number of monocytes and their subsets has a predictive value in term of clinical response to adalimumab and methotrexate in RA patients [38]. Infliximab treatment also induced an increase in intermediate monocytes, which produce high levels of TNF-α in response to LPS and show phagocytic capacity, thus behaving as proinflammatory cells [39]. Here we describe variations in the percentages of monocytes and in their subsets in patients treated with different anti-TNF-α drugs. The percentage of total monocytes, *i*.*e*., all CD14+ cells, as well as the percentage of classical monocytes in patients treated with adalimumab was significantly lower if compared to patients treated with etanercept and infliximab. However, the stratification of patients according to PASI did not reveal any difference in the percentage of monocyte subsets between R and NR to the different treatments. The loss of monocytes in patients treated with adalimumab could be caused by three, non-mutually exclusive phenomena, *i*.*e*., redistribution and homing of cells, increased differentiation (*e*.*g*., towards osteoclasts), or net loss of cells due to apoptosis. If on the one side, it is difficult to analyze monocyte redistribution to tissues other than blood, or to detect osteoclastic differentiation, on the other side, it is possible to hypothesize that, as already demonstrated using *in vitro* models [40], these cells are sensitive to a pro-apoptotic action of the drug. Regarding non-classical monocytes, while their percentage did not vary among different groups, the expression of HLA-DR was higher in non-classical monocytes from patients treated with infliximab than non-classical monocytes from patients treated with etanercept. It is well known that the expression of MHC class II molecules is directly correlated to the capacity to present antigens. Thus, it is possible to envisage that anti-TNF drugs are also able, to a certain extent, to modulate this fundamental function of immune cells.

In summary, our study suggests that a complex remodelling of the TNF-α/sTNFR system occurs in patients with psoriasis treated with anti-TNF-α drugs. Other studies are, however, needed to further characterize functional changes of monocytes in patients treated with different drugs, and that could devote their attention to other early, crucial aspects of inflammatory phenomena modulated by these drugs. Therefore, the correlations between plasma levels of TNF-α, its soluble receptor, the phenotype of monocytes and their ability to produce cytokines must be investigated also in other human diseases that benefit of he treatment with molecules that block TNF-α.
